# Oral rehabilitation and quality of life in head and neck cancer patients receiving dental clearance prior to radiotherapy: a retrospective observational study

**DOI:** 10.1007/s00520-025-09879-8

**Published:** 2025-09-11

**Authors:** Matthijs in ’t Veld, Frank K. J. Leusink, Chayenne N. Chhangur, Naichuan Su, Femke Jansen, Marije R. Vergeer, Irma M. Verdonck-de Leeuw, Engelbert A. J. M. Schulten

**Affiliations:** 1 Department of Oral and Maxillofacial Surgery/Oral Pathology, Amsterdam UMC and Academic Centre for Dentistry Amsterdam (ACTA), Vrije Universiteit Amsterdam, De Boelelaan 1117, 1081 HV Amsterdam, The Netherlands; 2 Department of Oral Public Health, Academic Centre for Dentistry Amsterdam, University of Amsterdam and Vrije Universiteit Amsterdam, Gustav Mahlerlaan 3004, 1081 LA Amsterdam, The Netherlands; 3 Department Otolaryngology-Head and Neck Surgery, Vrije Universiteit Amsterdam, De Boelelaan 1117, 1081 HV Amsterdam, The Netherlands; 4 Cancer Center Amsterdam Research Institute, Amsterdam UMC, Vrije Universiteit Amsterdam, De Boelelaan 1117, 1081 HV Amsterdam, The Netherlands; 5 Department of Radiation Oncology, Amsterdam UMC, De Boelelaan 1117, 1081 HV Amsterdam, The Netherlands

**Keywords:** Head and neck cancer, Oral cancer, Quality of life, Oral rehabilitation

## Abstract

**Purpose:**

This retrospective observational study examined oral rehabilitation and the total oral rehabilitation time (TORT) in head and neck cancer patients (HNC) who underwent full dental clearance before intensity-modulated radiotherapy (IMRT). Additionally, it assessed changes in health-related quality of life (HRQoL) before and after IMRT and oral rehabilitation.

**Methods:**

HNC patients with HRQol data who underwent full dental clearance before IMRT in Amsterdam UMC between 2008 and 2021 were included. The EORTC QLQ-C30 and QLQ-H&N35 were used to assess HRQoL before dental clearance at baseline (*T*_0_), after dental clearance and IMRT (*T*_1_), after oral rehabilitation with conventional dentures (*T*_2_), and after oral rehabilitation with implant-retained overdentures (*T*_3_).

**Results:**

HRQoL data were available for 28 patients at *T*_0_, *T*_1_, and *T*_2_; all received conventional dentures (TORT 13.5 months; range 4.8–64.0). Five also received implant-retained overdentures (TORT 29.8 months; range 25.8–31.5). Role and cognitive functioning declined at *T*_1_ vs. *T*_0_ (*p* < 0.01, *p* = 0.01), while emotional functioning improved (*p* < 0.01). HRQoL symptoms increased significantly at *T*_1_ vs. *T*_0_, including taste and smell, social contact troubles, and dry mouth. At *T*_2_, oral pain, supplement use, and weight gain decreased significantly (*p* = 0.05, *p* = 0.02, *p* < 0.01), while teeth and financial problems (*p* = 0.01, *p* = 0.04) increased compared to *T*_1_.

**Conclusion:**

All HNC patients in this study underwent dental clearance before IMRT and received oral rehabilitation with conventional dentures, with a mean TORT of over a year. Patients after oral rehabilitation had less oral pain but more problems with teeth and finances. Large prospective cohort studies are needed to confirm these findings.

## Introduction

Head and neck cancer (HNC) was the seventh most prevalent cancer worldwide in 2020, with an estimated 878,348 new cases and 444,347 associated fatalities [1]. HNC involves malignancies in the upper respiratory and digestive tract, including the oral cavity, the pharynx, and the larynx.

Treatment modalities for HNC include surgery with or without adjuvant radiotherapy (RT) or chemoradiation (CRT), primary RT or primary CRT [2]. The current standard in RT, intensity modulated RT (IMRT), has been successful in decreasing radiation doses to adjacent organs at risk [3]. However, acute and long-term side-effects have been reported, including hyposalivation, trismus, dysphagia, infection, mucositis, and radiation caries [4, 5]. RT also significantly increases the risk of developing osteoradionecrosis of the jaw as a serious, long-term complication [5]. To reduce this risk and allow adequate wound healing, complete elimination of existing dental foci is recommended 10 to 14 days prior to RT with an expected cumulative radiation dose of ≥ 40 Gy in the head and neck region [6, 7]. For this purpose, some patients undergo full dental clearance and require oral rehabilitation afterwards. Dental extractions in HNC patients are frequently performed to eliminate dental foci prior to radiotherapy, thereby reducing the risk of osteoradionecrosis. However, in surgically treated cases, extractions may also be required due to the extent of the planned oncologic resection. For instance, in the context of a segmental mandibulectomy, dentition located within the surgical field must be removed irrespective of its condition. These surgical indications for dental extraction—unrelated to oral health status—highlight the heterogeneity in treatment needs and clinical outcomes among HNC patients. To minimize this variability, the present study focuses on a more homogenous patient population undergoing full dental clearance prior to IMRT alone, without major ablative surgery.

Multiple treatment modalities for oral rehabilitation exist, including full conventional dentures (CDs), implant-retained overdentures (IODs), or fixed prosthetics on dental implants. Whereas CDs can be placed within four to five months after the last radiation dose, dental implant placement is recommended after six to eight months post-RT [8]. Although studies have shown high success rates for immediately loaded dental implants in the mandible, conventional implant loading protocols recommend an additional osseo-integration period of 3 to 6 months [2, 9]. As a result, HNC patients often undergo a long total oral rehabilitation time (TORT), potentially leading to a decrease in health-related quality of life (HRQoL).

In order to improve the quality of oral health care in HNC patients undergoing RT, more data regarding HRQoL during the course of the treatment is required. The existing literature suggests that HRQoL is negatively affected by tooth loss [10, 11]. Studies have also reported that tooth loss is associated with impaired oral function and reduced social and psychological well-being [11]. Furthermore, studies have suggested an association between oral rehabilitation and an increased HRQoL in non-HNC patients [12, 13]. Recent studies have reported high success rates for immediately placed dental implants during ablative surgery, resulting in a shortened TORT [2, 14–17].

However, limited studies have examined changes in HRQoL of HNC patients undergoing RT from baseline after full dental clearance to post-RT and post-oral rehabilitation [18, 19]. For these patients, an increased risk of osteoradionecrosis of the jaw and the further impact of RT on oral functioning are considered challenging factors in oral rehabilitation. In addition, there is no clear consensus in the literature regarding the treatment strategy in the scope of oral rehabilitation. Therefore, this study is aimed at describing the various types of oral rehabilitation utilized in HNC patients and assessing their HRQoL from the initiation of treatment (prior to dental clearance and RT) to the completion of oral rehabilitation. We hypothesized (1) that HRQoL would decrease after full dental clearance and RT and (2) that HRQoL would improve after oral rehabilitation. This research holds importance in optimizing patient care for individuals undergoing full dental clearance and RT for HNC.

## Material and methods

This retrospective observational cohort study was conducted with the approval of the Medical Ethical Review Committee of the VU University Medical Center (VUmc), which was obtained in September 2019 (registration number: 2019.253). This study was reported in accordance with the Strengthening the Reporting of Observational Studies in Epidemiology (STROBE) guideline [20].

### Study population

The study population consisted of HNC patients who received full dental clearance during panendoscopy prior to RT in the Department of Oral and Maxillofacial Surgery/Oral Pathology, Amsterdam UMC, location VUmc, The Netherlands in the period 2008–2021.

Patients were considered eligible for participation, when meeting the following criteria: (1) age ≥ 18 years, (2) confirmed diagnosis of HNC, (3) referred to the Department of Oral and Maxillofacial Surgery/Oral Pathology for dental screening, (4) full dental clearance prior to RT, (5) treated by IMRT as a standard method of care, (6) received oral rehabilitation with a CD, and (7) data of the EORTC QLQ-C30 and EORTC QLQH&N35 questionnaire were both available on multiple moments during the course of treatment.

Patients were excluded from the study in case of (1) explicit declaration to not use their data in scientific research (i.e., letter of objection filed in the medical record), (2) no full dental clearance before RT, and (3) maxillary or mandibular resection with or without reconstruction with microvascular free flaps.

Screening for eligibility took place by two independent researchers (MV and CC), based on the inclusion and exclusion criteria. Disagreements were resolved by discussion. When consensus was not achieved, a third party was consulted (FL).

### Setting

**Fig. 1:**
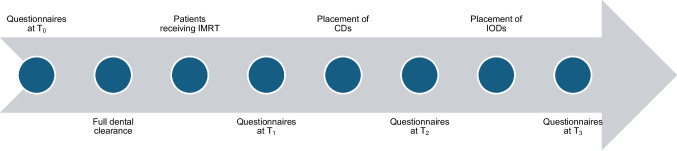
Timeline of quality of life measurements across treatment phases. CDs, conventional dentures; IODs, implant-retained overdenture; IMRT, intensity-modulated radiotherapy; *T*_0_, before dental clearance; *T*_1_, after radiotherapy; *T*_2_, after placement of CDs; *T*_3_, after placement of IODs

Since 2008, all HNC patients who visit the Department of Otolaryngology-Head and Neck Surgery and Radiotherapy are asked to complete the two HRQoL questionnaires during treatment and follow-up as part of clinical practice. The moments of HRQoL measurement were identified and categorized as follows (see also Fig. 1):

- *T*_0_ = before dental clearance
- *T*_1_ = after RT, before oral rehabilitation
- *T*_2_ = after placement of CDs (oral rehabilitation)
- *T*_3_ = after placement of IODs (oral rehabilitation)

The time interval between *T*_0_ and *T*_3_ varied considerably between patients, depending on individual recovery, severity of radiotherapy-related side effects, and logistical factors such as treatment planning and appointment availability. Data regarding study group characteristics (e.g., gender, age, tumor site, tumor stage, and occlusal units) were retrieved from the medical database (EPIC® Hyperspace, Verona, Wisconsin, USA) in Amsterdam UMC.

### Patient reported outcome measures

The Dutch versions of the EORTC QLQ-C30 (version 3.0) and the EORTC QLQ-H&N35 questionnaires were used to collect HRQoL data at *T*_0_, *T*_1,_ *T*_2_ and *T*_3_. The EORTC QLQ-C30 is a reliable and valid measure for HRQoL in HNC patients [21]. Supplementary to this, the EORTC QLQ-H&N35 offers a reliable measurement of HRQoL before, during, and after treatment of HNC [22]. The EORTC QLQ-C30 consists of a global health status GHS/HRQoL scale, five functional scales (physical, role, cognitive, emotional, and social functioning), three multi-item symptom scales (fatigue, pain, nausea/vomiting), and six single-item symptom scales (dyspnea, insomnia, appetite loss, constipation, diarrhea, and financial difficulties). The EORTC QLQ-H&N35 comprises 35 single- and multi-item symptom scales, including oral pain, swallowing, senses problems, speech problems, social eating, social contact, sexuality, teeth, opening mouth, dry mouth, sticky saliva, coughing, felt ill, painkillers, nutritional supplements, feeding tube, weight loss, and weight gain.

Raw scores for both questionnaires were converted to 0–100 scale scores. For the QLQ-C30, higher scores on GHS/HRQoL and functional scales indicated better HRQoL and functioning, whereas higher scores on symptom scales reflected more severe symptoms. Similarly, higher QLQ-H&N35 scores represented more severe symptoms.

### Statistical analysis

Baseline characteristics of the groups were presented using descriptive statistics. A Shapiro–Wilk test was used to assess normality for each scale score of the two questionnaires. Normally distributed continuous variables were reported as means and 95% confidence intervals or standard deviations. Variables that were not normally distributed were reported as medians with 25th and 75th percentiles or minimum and maximum. Categorical variables were reported as number and percentage. *P*-values less than 0.05 were considered statistically significant. Descriptive statistics were performed using SPSS version 28.0 (IBM Corp, Armonk, NY, USA). The assessment of change of scale scores for each aspect of HRQoL over time (i.e., *T*_0_, *T*_1_, and *T*_2_) was conducted with linear mixed models (LMM) analysis with random intercept in R version 4.3.3 (Posit Software, Boston, MA, USA).

## Results

### Study population

**Fig. 2:**
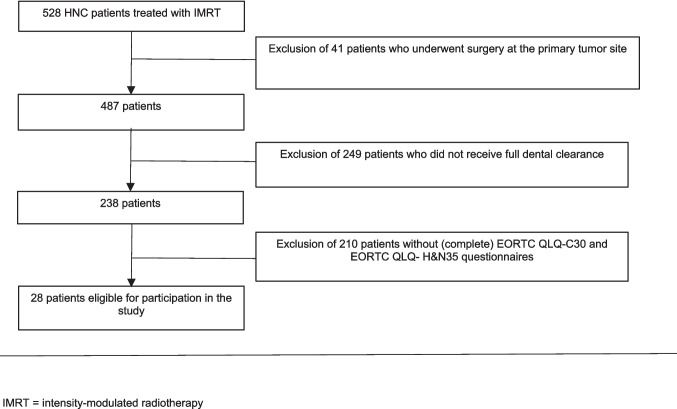
Flowchart of the selection procedure. IMRT, intensity-modulated radiotherapy

In total, 528 HNC patients who received RT in Amsterdam UMC, location VUmc, between 2008 and 2021 were retrospectively screened for eligibility. Eventually, 28 patients were included for final analysis. In Table 1, the demographic and clinical characteristics of the study population are shown, including both the patients included in the final analysis (i.e., the patients who filled out the questionnaires) and those who were excluded from the analysis (i.e., those who underwent dental clearance but did not complete the questionnaires), to explore potential differences between the two cohorts. Based on Table 1, some baseline variables may have moderate difference between the two cohorts of patients, such as tumor site and nodal stage, which indicates that the patients included in the final analysis may not be very well representative to the target population. In Fig. 2, the selection of the study population is shown.

**Table 1:** Demographic and clinical characteristics of the included and excluded head and neck cancer patients

Number of patients	*N* = 28	*N* = 210
Gender	Number (percentage)	
Male	19 (67.9%)	164 (78.1%)
Female	9 (32.1%)	46 (21.9%)
Age at dental clearance (in years)		
Median (IQR)	68.5 (15.5)	66.17 (12.0)
Minimum	50	40
Maximum	87	91
Tumor site	Number (percentage)	
Oropharynx	17 (60.7%)	77 (36.7%)
Larynx	4 (14.3%)	37 (17.6)
Hypopharynx	5 (17.9%)	39 (18.6%)
Oral cavity carcinoma	2 (7.1%)	39 (18.6)
Unknown primary tumor	0 (0.0%)	8 (3.8%)
Salivary gland tumor	0 (0.0%)	5 (2.4%)
Missing data	0 (0.0%)	5 (2.4%)
Tumor stage	Number (percentage)	
T1	5 (7.9%)	21 (10.0%)
T2	9 (32.1%)	60 (28.6%)
T3	7 (25.0%)	44 (21.0%)
T4	7 (25.0%)	74 (35.2%)
Tx	0 (0.0%)	6 (2.9%)
Missing data	0 (0.0%)	5 (2.4%)
Nodal stage	Number (percentage)	
N0	9 (32.1%)	89 (42.4%)
N1	4 (14.3%)	23 (11.0%)
N2	15 (53.6%)	83 (40.3%)
N3	0 (0.0%)	10 (4.8%)
Missing data	0	5 (2.4%)
Occlusal units before dental clearance		
Median (IQR)	1 (3)	
Minimum	0	
Maximum	10	
Time between dental clearance and prosthetic rehabilitation with conventional dentures (in months)		
Mean (95% CI)	13.5 (8.8–18.5)	
Minimum	4.8	
Maximum	64	
Time between dental clearance and prosthetic rehabilitation with dental implants (in months)		
Mean (95% CI)	29.8 (27–32.5)	
Minimum	25.8	
Maximum	31.5	

*95% CI*, 95% confidence interval; *IQR*, interquartile range

### Oral rehabilitation

The time interval between dental clearance and the placement of CDs had a mean duration of 13.5 months, with a range of 4.8 to 64 months, while the mean TORT between RT and the placement of CDs was 11.5 months. Among the 28 patients who received CDs, five (19%) eventually opted for IODs, resulting in an average duration between dental clearance and IOD placement of 29.8 months, ranging from 25.8 to 31.5 months. The average duration between completion of *T*_0_ HRQoL questionnaires and full dental clearance was 1.2 weeks (range 0–5), and the average time from full dental clearance to the initiation of RT was 2.7 weeks (range 1–10). The mean duration of RT was 1.3 months (range 0.8–1.5), and the average interval between the completion of RT and *T*_1_ was 2.8 months (range 0.8–16). The average time between the completion of RT and completion of *T*_2_ HRQoL instruments was 24.8 weeks (range 3.8–57), while the mean time between the placement of the prosthesis and *T*_2_ was 16.5 weeks (range 0.8–37.5).

### Health-related quality of life

Mean scores, standard deviations, medians, and 25th and 75th quartiles of the EORTC QLQ-C30 and HN35 are shown in Tables 2 and 3, respectively. There was a significant impact of IMRT on HRQOL. The EORTC QLQ-C30 presented a statistically significant decrease between *T*_1_ and *T*_0_ for role functioning (β = − 0.92, *p* = 0.04) and cognitive functioning (β = − 0.80, *p* = 0.01). Patients reported significantly better emotional functioning at *T*_1_ than at *T*_0_ (β = 1.21, *p* = 0.03).

**Table 2:** Quality of life among head and neck cancer patients at different time points as measured by the EORTC QLQ-C30 using linear mixed model analysis

		Median scores (Q1–Q3)	Mean scores (SD)	Regression coefficient between T1 and T0 (ref.)	*p*-value (T1 vs. T0)	regression coefficient between T2 and T1 (ref.)	*p*-value (T2 vs. T1)
Global health status^a^	T0	58.33 (*n* = 18) (50.00–83.33)	64.35 (18.70)	Ref			
	T1	66.67 (*n* = 23) (58.33–83.33)	70.65 (14.62)	0.80 (− 0.87–2.47)	0.35 (*n* = 15)	Ref	
	T2	75.00 (*n* = 24) (50.00–83.33)	68.40 (24.07)			− 0.51 (− 2.03–1.02)	0.51 (*n* = 20)
Functional scales^a^							
Physical functioning	T0	90.00 (*n* = 18) (60.00–95.00)	82.96 (17.30)	Ref			
	T1	73.33 (*n* = 23) (66.67–86.67)	74.20 (22.50)	− 1.29 (− 2.66–0.09)	0.07 (*n* = 15)	Ref	
	T2	80.00 (*n* = 25) (66.67–93.33)	75.20 (20.14)			0.036 (− 1.21–1.28)	0.95 (*n* = 20)
Role functioning	T0	100.00 (*n* = 18) (66.67–100.00)	87.04 (20.26)	Ref			
	T1	66.67 (*n* = 23) (50.00–100.00)	**72.46** **(28.25)**	** − 0.92** **(− 1.77 – − 0.07)**	**0.04** **(*n* = 15)**	Ref	
	T2	66.67 (*n* = 25) (50.00–100.00)	71.33 (25.24)			− 0.06 (− 0.83–0.72)	0.88 (*n* = 20)
Emotional functioning	T0	70.83 (*n* = 18) (62.50–83.33)	68.52 (19.08)	Ref			
	T1	83.33 (*n* = 23) (66.67–100.00)	81.88 (18.74)	1.21 (0.16–2.26)	0.03 (*n* = 15)	Ref	
	T2	83.33 (*n* = 25) (66.67–100.00)	75.67 (27.63)			− 0.32 (− 1.27–0.63)	0.51 (*n* = 20)
Cognitive functioning	T0	100.00 (*n* = 18) (83.33–100.00)	94.44 (9.90)	Ref			
	T1	83.33 (*n* = 23) (66.67–100.00)	**81.16** **(19.66)**	** − 0.80** **(− 1.34 – − 0.27)**	**0.01** **(*n* = 15)**	Ref	
	T2	83.33 (*n* = 25) (66.67–100.00)	80.00 (22.57)			0.02 (− 0.47–0.50)	0.94 (*n* = 20)
Social functioning	T0	100.00 (*n* = 18) (100.00–100.00)	93.52 (17.28)	Ref			
	T1	91.67 (*n* = 22) (62.50–100.00)	82.58 (22.11)	− 0.65 (− 1.38–0.07)	0.08 (*n* = 14)	Ref	
	T2	100.00 (*n* = 25) (75.00–100.00)	85.33 (23.23)			0.20 (− 0.46–0.86)	0.56 (*n* = 19)
Symptom scales^b^							
Fatigue	T0	22.22 (*n* = 18) (8.33–36.11)	23.46 (18.23)	Ref			
	T1	33.33 (*n* = 23) (0.00–44.44)	28.99 (26.11)	0.52 (− 0.51–1.56)	0.32 (*n* = 15)	Ref	
	T2	33.33 (*n* = 25) (5.56–52.78)	32.67 (25.17)			0.45 (− 0.48–1.39)	0.34 (*n* = 20)
Nausea and vomiting	T0	0.00 (*n* = 18) (0.00–16.67)	11.11 (22.14)	Ref			
	T1	0.00 (*n* = 23) (0.00–0.00)	10.87 (23.36)	0.03 (− 0.77–0.83)	0.94 (*n* = 15)	Ref	
	T2	0.00 (*n* = 25) (0.00–0.00)	11.33 (23.92)			0.05 (− 0.68–0.77)	0.90 (*n* = 20)
Pain	T0	16.67 (*n* = 18) (0.00–33.33)	21.30 (25.44)	Ref			
	T1	16.67 (*n* = 23) (0.00–33.33)	23.19 (25.50)	0.13 (− 0.69–0.96)	0.75 (*n* = 15)	Ref	
	T2	16.67 (*n* = 25) (0.00–50.00)	23.33 (27.64)			0.03 (− 0.73–0.78)	0.94 (*n* = 20)
Dyspnea	T0	16.67 (*n* = 18) (0.00–33.33)	20.37 (23.26)	Ref			
	T1	0.00 (*n* = 23) (0.00–33.33)	21.74 (25.84)	0.13 (− 0.27–0.53)	0.53 (*n* = 15)	Ref	
	T2	0.00 (*n* = 25) (0.00–33.33)	21.33 (27.01)			0.03 (− 0.34–0.39)	0.88 (*n* = 20)
Insomnia	T0	16.67 (*n* = 18) (0.00–66.67)	29.63 (34.09)	Ref			
	T1	33.33 (*n* = 23) (0.00–66.67)	30.44 (30.01)	0.02 (− 0.38–0.42)	0.92 (*n* = 15)	Ref	
	T2	0.00 (*n* = 25) (0.00–66.67)	24.00 (29.69)			− 0.11 (− 0.47–0.25)	0.55 (*n* = 20)
Loss of appetite	T0	0.00 (*n* = 18) (0.00–8.33)	12.96 (28.33)	Ref			
	T1	33.33 (*n* = 23) (0.00–66.67)	27.54 (31.22)	0.45 (− 0.16–1.05)	0.15 (*n* = 15)	Ref	
	T2	0.00 (*n* = 25) (0.00–66.67)	32.00 (41.37)			0.13 (− 0.41–0.68)	0.63 (*n* = 20)
Constipation	T0	0.00 (*n* = 18) (0.00–0.00)	3.70 (15.71)	Ref			
	T1	0.00 (*n* = 23) (0.00–0.00)	11.59 (25.84)	0.24 (− 0.12–0.60)	0.19 (*n* = 15)	Ref	
	T2	0.00 (*n* = 25) (0.00–0.00)	5.33 (15.75)			− 0.18 (− 0.51–0.15)	0.29 (*n* = 20)
Diarrhea	T0	0.00 (*n* = 18) (0.00–0.00)	14.81 (20.52)	Ref			
	T1	0.00 (*n* = 23) (0.00–33.33)	14.49 (28.12)	− 0.05 (− 0.44–0.34)	0.78 (*n* = 15)	Ref	
	T2	0.00 (*n* = 25) (0.00–44.44)	13.33 (21.52)			− 0.01 (− 0.37–0.35)	0.95 (*n* = 20)
Financial problems	T0	0.00 (*n* = 18) (0.00–0.00)	9.26 (25.06)	Ref			
	T1	0.00 (*n* = 22) (0.00–8.33)	9.09 (18.35)	0.10 (− 0.27–0.47)	0.60 (*n* = 14)	Ref	
	T2	0.00 (*n* = 25) (0.00–33.33)	**24.00 (29.69)**			**0.36 (0.03–0.70)**	**0.04** **(*n* = 19)**

*EORTC*, European Organization for Research and Treatment of Cancer; *SD*, standard deviation. Data are in median, quartiles, mean, and SD. *T*_0_ = before dental clearance. *T*_1_ = after radiotherapy. *T*_2_ = after receiving conventional dentures. A higher score indicates more symptoms. Bold printing indicates *p* < 0.05. ^a^Higher scores reflect better HRQoL/functioning. ^b^Higher scores reflect more severe symptoms

**Table 3:** EORTC QLQ-H&N35 measured quality of life among head and neck cancer patients at different time points using linear mixed model analysis

		Median scores (Q1–Q3)	Mean scores (SD)	Regression coefficient between T1 and T0 (ref.)	*p*-value (T1 vs. T0)	Regression coefficient between T2 and T1 (ref.)	*p*-value (T2 vs. T1)
Symptom scales^a^							
Oral pain	T0	25.00 (*n* = 18) (0.00–45.83)	27.78 (28.87)	Ref			
	T1	33.33 (*n* = 23) (0.00–66.67)	37.32 (32.36)	1.35 (− 0.50–3.20)	0.16 (*n* = 15)	Ref	
	T2	16.67 (*n* = 25) (0.00–37.50)	22.33 (24.62)			** − 1.76** **(− 3.43 – − 0.08)**	**0.05** **(*n*** **= 20)**
Swallowing	T0	25.00 (*n* = 18) (0.00–68.75)	34.74 (32.87)	Ref			
	T1	33.33 (*n* = 23) (0.00–66.67)	36.59 (33.59)	0.89 (− 0.85–2.63)	0.32 (*n* = 15)	Ref	
	T2	16.67 (*n* = 25) (0.00–33.33)	24.56 (29.22)			− 1.39 (− 2.96–0.18)	0.09 (*n* = 20)
Senses problems (taste/smell)	T0	0.00 (*n* = 18) (0.00–16.67)	11.11 (22.87)	Ref			
	T1	33.33 (*n* = 23) (16.67–50.00)	**30.43** (25.45)	**1.20** **(0.42–1.99)**	** < 0.01** **(*n*** **= 15**)	Ref	
	T2	16.67 (*n* = 25) (0.00–83.33)	34.00 (37.42)			0.00 (− 0.71–0.71)	0.10 (*n* = 20)
Speech problems	T0	11.11 (*n* = 18) (0.00–33.33)	19.75 (22.72)	Ref			
	T1	22.22 (*n* = 23) (0.00–33.33)	22.71 (22.10)	0.62 (− 1.27–0.63)	0.34 (*n* = 15)	Ref	
	T2	11.11 (*n* = 25) (0.00–44.44)	25.44 (30.68)			0.14 (− 1.01–1.29)	0.81 (*n* = 20)
Trouble with social eating	T0	4.17 (*n* = 18) (0.00–29.17)	20.83 (31.34)	Ref			
	T1	25.00 (*n* = 22) (12.50–52.08)	34.47 (29.91)	1.76 (− 0.09–3.62)	0.07 (*n* = 15)	Ref	
	T2	16.67 (*n* = 25) (0.00–50.00)	30.00 (34.86)			− 0.38 (− 2.06–1.30)	0.66 (*n* = 19)
Trouble with social contact	T0	0.00 (*n* = 18) (0.00–13.33)	5.19 (7.43)	Ref			
	T1	8.33 (*n* = 23) (0.00–20.00)	14.57 (19.11)	**2.04** **(0.54–3.54)**	**0.01** **(*n*** **= 15)**	Ref	
	T2	0.00 (*n* = 25) (0.00–30.00)	16.00 (24.72)			− 0.31 (− 1.66–1.05)	0.65 (*n* = 20)
Less sexuality	T0	0.00 (*n* = 13) (0.00–25.00)	14.10 (22.41)	Ref			
	T1	33.33 (*n* = 19) (0.00–50.00)	30.70 (29.01)	0.90 (− 0.05–1.84)	0.07 (*n* = 9)	Ref	
	T2	16.67 (*n* = 20) (0.00–33.33)	25.83 (32.66)			− 0.54 (− 1.29–0.22)	0.17 (*n* = 14)
Symptom items^b^							
Teeth	T0	0.00 (*n* = 18) (0.00–33.33)	12.96 (23.26)	Ref			
	T1	0.00 (*n* = 18) (0.00–0.00)	7.41 (18.28)	− 0.29 (− 0.96–0.38)	0.40 (*n* = 12)	Ref	
	T2	33.33 (*n* = 22) (0.00–100.00)	42.42 (42.64)			**0.9** **(0.28–1.51)**	**0.01** **(***n*** = 14)**
Opening mouth	T0	0.00 (*n* = 23) (0.00–0.00)	11.11 (28.01)	Ref			
	T1	0.00 (*n* = 23) (0.00–33.33)	23.19 (30.87)	0.42 (− 0.00–0.83)	0.06 (*n* = 15)	Ref	
	T2	0.00 (*n* = 25) (0.00–66.67)	28.00 (35.59)			0.26 (− 0.12–0.64)	0.18 (*n* = 20)
Dry mouth	T0	0.00 (*n* = 18) (0.00–33.33)	11.11 (16.17)	Ref			
	T1	33.33 (*n* = 23) (0.00–100.00)	46.38 (38.58)	**1.04** **(0.52–1.55)**	** < 0.01** **(*n* = 15)**	Ref	
	T2	33.33 (*n* = 25) (0.00–66.67)	36.00 (34.59)			− 0.26 (− 0.73–0.21)	0.28 (*n* = 20)
Sticky saliva	T0	0.00 (*n* = 18) (0.00–33.33)	20.37 (28.33)	Ref			
	T1	50.00 (*n* = 22) (0.00–100.00)	45.45 (43.09)	**0.80** **(0.21–1.40)**	**0.01** **(*n* = 15)**	Ref	
	T2	33.33 (*n* = 25) (0.00–66.67)	38.67 (40.46)			− 0.10 (− 0.64–0.43)	0.71 (*n* = 19)
Coughing	T0	33.33 (*n* = 18) (0.00–66.67)	42.60 (33.93)	Ref			
	T1	33.33 (*n* = 23) (0.00–33.33)	31.88 (30.94)	− 0.20 (− 0.64–0.24)	0.37 (*n* = 15)	Ref	
	T2	33.33 (*n* = 25) (0.00–66.67)	29.33 (33.77)			0.009 (− 0.39–0.40)	0.96 (*n* = 20)
Felt ill	T0	0.00 (*n* = 18) (0.00–41.67)	18.52 (28.52)	Ref			
	T1	0.00 (*n* = 23) (0.00–33.33)	15.94 (22.18)	− 0.03 (− 0.47–0.40)	0.88 (n = 15)	Ref	
	T2	0.00 (*n* = 25) (0.00–33.33)	20.00 (27.22)			0.13 (− 0.27–0.52)	0.53 (*n* = 20)
Pain killers	T0	50.00 (*n* = 18) (0.00–100.00)	50.00 (51.50)	Ref			
	T1	100.00 (*n* = 23) (0.00–100.00)	56.52 (50.69)	0.05 (− 0.25–0.34)	0.76 (*n* = 15)	Ref	
	T2	0.00 (*n* = 25) (0.00–100.00)	44.00 (50.66)			− 0.13 (− 0.40–0.14)	0.36 (*n* = 20)
Nutritional supplements	T0	0.00 (*n* = 18) (0.00–0.00)	11.11 (32.33)	Ref			
	T1	100.00 (*n* = 22) (100.00–100.00)	81.82 (39.48)	**0.71** **(0.44–0.98)**	** < 0.01** **(*n*** **= 14)**	Ref	
	T2	100.00 (*n* = 25) (0.00–100.00)	**52.00** **(50.99)**			** − 0.30** **(− 0.55 – − 0.05)**	**0.02** **(*n* = 19)**
Feeding tube	T0	0.00 (*n* = 18) (0.00–0.00)	**0.00** **(0.00)**	Ref			
	T1	0.00 (*n* = 23) (0.00–100.00)	**39.13** **(49.90)**	**0.40** **(0.19–0.61)**	** < 0.01** **(n = 14)**	Ref	
	T2	0.00 (*n* = 25) (0.00–50.00)	24.00 (43.59)			− 0.14 (− 0.33–0.05)	0.16 (*n* = 20)
Weight loss	T0	0.00 (*n* = 18) (0.00–100.00)	33.33 (48.51)	Ref			
	T1	0.00 (*n* = 23) (0.00–100.00)	30.43 (47.05)	− 0.03 (− 0.30–0.24)	0.85 (*n* = 15)	Ref	
	T2	0.00 (*n* = 25) 0.00–0.00	20.00 (40.82)			− 0.10 (− 0.35–0.14)	0.41 (*n* = 20)
Weight gain	T0	0.00 (*n* = 18) (0.00–0.00)	11.11 (32.33)	Ref			
	T1	100.00 (*n* = 23) (0.00–100.00)	**60.87** **(49.90)**	**0.46** **(0.23–0.69)**	** < 0.01** **(*n*** **= 15)**	Ref	
	T2	0.00 (*n* = 25) 0.00–0.00	**20.00** **(40.82)**			** − 0.41** **(− 0.62 – − 0.20)**	** < 0.01** **(***n* **= 20**)

T_0_ = before dental clearance. T_1_ = after radiotherapy. T_2_ = after receiving conventional dentures. A higher score indicates more symptoms. Bold printing indicates *p* < 0.05. ^a^Higher scores reflect more severe symptoms

Based on the EORTC QLQ-H&N35, patients reported significantly higher levels of social contact problems (β = 2.04, *p* = 0.01) and disturbances with taste and smell at *T*_1_ compared to *T*_0_ (β = 1.20, *p* < 0.01). Moreover, there was a significant increase in symptoms at *T*_1_ compared to *T*_0_ for dry mouth (β = 1.04, *p* < 0.01) and sticky saliva (β = 0.80, *p* = 0.01). Significant increases in weight gain (β = 0.46, *p* < 0.01), nutritional supplements use (β = 0.71, *p* < 0.01*)*, and tube feeding (β = 0.40, *p* < 0.01) were reported at *T*_1_ compared to *T*_0_.

Comparing *T*_2_ with *T*_1_, patients reported significant decreased scores regarding oral pain (β = − 1.76, *p* = 0.05), use of nutritional supplements (β = − 0.30, *p* = 0.02), and weight gain (β = − 0.41, *p* < 0.01). However, patients reported significant more problems with their teeth (β = 0.9, *p* = 0.01) and financial problems (β = 0.36, *p* = 0.04) after placement of CDs.

Because there were too many missing data, the results at *T*_3_, after oral rehabilitation with IODs, were not statistically analyzed and are, therefore, presented as descriptive statistics. Values are presented in means. Mean scores of emotional functioning slightly decreased at *T*_3_ (72.22 versus 75.67) compared to *T*_2_. Patients also reported decreased mean scores of role functioning (61.11 versus 71.33) and cognitive functioning (66.67 versus 80.00) at *T*_3_ compared to *T*_2_. Also, patients experienced more financial problems (33.33 versus 24.00) after placement of IODs than after placement of CDs and more problems with smell and taste (44.44 versus 34.80). Additionally, patients reported lower scores for sticky saliva at *T*_3_ (33.33) in contrast to *T*_2_ (38.67) and *T*_1_ (45.45). Other scoring items, medians, mean scores, ranges, and standard deviations of the EORTC QLQ-C30 and the EORTC QLQ-H&N35 questionnaires are shown in Table 4.

**Table 4:** Descriptive calculated scores of EORTC QLQ-C30 and H&N35 scales for head and neck cancer patients at T_3_

Scales	Median (range)	Mean (SD)	*N*
*QLQ-C30*			
*Global health status/HRQoL*^*a*^	66.67 (33.33–75.00)	58.33 (22.05)	3
*Functional scales*			
*Physical functioning*	73.33 (53.33–93.33)	73.33 (20.00)	3
*Role functioning*	50.00 (50.00–83.33)	61.11 (19.25)	3
*Emotional functioning*	83.33 (41.67–91.67)	72.22 (26.78)	3
*Cognitive functioning*	66.67 (66.67–66.67)	66.67 (0.00)	3
*Social functioning*	66.67 (66.67–83.33)	72.22 (9.62)	3
*Symptom scales*^*b*^			
*Fatigue*	33.33 (33.33–55.56)	40.74 (12.83)	3
*Nausea and vomiting*	0.00 (0.00–16.67)	5.56 (9.62)	3
*Pain*	50.00 (33.33–83.33)	55.56 (25.46)	3
*Dyspnea*	0.00 (0.00–66.67)	22.22 (38.49)	3
*Insomnia*	66.67 (66.67–100.00)	77.78 (19.25)	3
*Loss of appetite*	33.33 (0.00–33.33)	22.22 (19.25)	3
*Constipation*	0.00 (0.00–66.67)	22.22 (38.49)	3
*Diarrhea*	0.00 (0.00–66.67)	22.22 (38.49)	3
*Financial problems*	0.00 (0.00–66.67)	33.33 (33.33)	3
*H&N35*			
*Symptom scales*^*a*^			
*Oral pain*	41.67 (25.00–50.00)	38.89 (12.73)	3
*Swallowing*	58.33 (0.00–66.67)	41.67 (36.32)	3
*Senses problems (taste/smell)*	50.00 (33.33–50.00)	44.44 (9.62)	3
*Speech problems*	11.11 (0.00–55.56)	22.22 (29.40)	3
*Trouble with social eating*	100.00 (0.00–100.00)	66.67 (57.74)	3
*Trouble with social contact*	26.67 (0.00–50.00)	25.56 (25.02)	3
*Less sexuality*	41.67 (16.67–66.67)	41.67 (35.36)	2
*Symptom items*^*b*^			
*Teeth*	66.67 (33.33–100.00)	66.67 (33.33)	3
*Opening mouth*	100.00 (0.00–100.00)	66.67 (57.74)	3
*Dry mouth*	66.67 (0.00–100.00)	55.56 (50.92)	3
*Sticky saliva*	0.00 (0.00–100.00)	33.33 (57.74)	3
*Coughing*	0.00 (0.00–33.33)	11.11 (19.25)	3
*Feeling ill*	33.33 (0.00–66.67)	33.33 (33.33)	3
*Painkillers*	100.00 (100.00–100.00)	100.00 (0.00)	3
*Nutritional supplements*	100.00 (0.00–100.00)	66.67 (57.74)	3
*Feeding tube*	0.00 (0.00–100.00)	33.33 (57.74)	3
*Weight loss*	0.00 (0.00–0.00)	0.00 (0.00)	3
*Weight gain*	0.00 (0.00–0.00)	0.00 (0.00)	3

*EORTC*, European Organization for Research and Treatment of Cancer; *SD*, standard deviation. Data are in mean, SD, median, minimum, and maximum. *T*_3_ = after receiving dental implants. A higher score indicates more symptoms. Descriptive statistics are shown at *T*_3_; no statistical analysis could be performed at *T*_3_, because of too many missing values. ^a^Higher scores reflect better HRQoL/functioning. ^b^Higher scores reflect more severe symptoms

## Discussion

In a cohort of 28 HNC patients who had undergone full dental clearance prior to RT and received CDs during oral rehabilitation, HRQoL was documented at four different timepoints. It was hypothesized that patients would experience a decrease in HRQoL after full dental clearance and RT and an increase in HRQoL after oral rehabilitation. Indeed, patients experienced a significant decline in role and cognitive functioning and reported significantly more problems with social contact, taste and smell, mouth opening, dry mouth, sticky saliva, use of nutritional supplements, tube feeding, and weight gain compared to before dental clearance and RT. However, patients reported significantly improved emotional functioning. Following the placement of CDs, patients reported more dental and financial problems compared to post-RT. However, they experienced less oral pain, reduced reliance on nutritional supplements, and less weight gain after receiving CDs than after RT.

In the present study, the mean time between completion of RT and the *T*_1_ questionnaire was approximately three months. Comparable to our findings, a retrospective observational study by Lal et al. reported an increase in dry mouth and sticky saliva in HNC patients 3 months after receiving IMRT [23]. An observational study by Loorents et al. reported an increase in fatigue, dry mouth, and sticky saliva in HNC patients 3 months after RT [24]. A study by Dahele et al. found a deterioration in all symptoms, except dry mouth, at the end of RT treatment and 6 months later, compared to pre-RT [25]. Most scores were comparable to our findings, except for global HRQoL, emotional functioning, dry mouth, problems with teeth, coughing, and illness, on which we found no significant change over time. In addition, our study shows that problems with sense of smell and taste, the use of nutritional supplements, and tube feeding were greater after RT than before dental clearance. Alterations in smell and taste are well known side effects of RT and likely result from localized damage to the chorda tympani and taste buds [26]. Radiation-induced fibrosis of healthy tissue (e.g., swallowing structures and vascular/neural/muscular damage) may cause long-term feeding-tube dependence and trismus [4, 27, 28]. Additionally, potentially also resulting from edentulous jaws, difficulties with eating, and the need for nutritional supplements may be present [29]. While the patients in our study reported weight gain after RT, previous studies among RT patients (both with and without total dental extraction) have reported the opposite [24, 30, 31]. Additionally, a recent study by Buurman et al. found that tooth extractions prior to RT are a risk factor for weight loss in patients with oropharyngeal squamous cell carcinoma [32]. A potential explanation for these contradictory results is that treatment protocols for pre-RT management and counseling of patients’ dietary intake may differ from one hospital to another. Such differences may underline the finding of Ravasco et al. that pre-RT dietary counseling had a positive impact on dietary intake 3 months post-RT [33]. Another possible explanation relates to the formulation of the weight gain question in the C30 questionnaire, which focuses on the past week. It is plausible that, in this cohort, patients initially experienced weight loss during a period for which we have no measurements but regained some weight shortly after treatment.

Patients in our study experienced significantly better emotional functioning (felt less tense, irritable and depressed in the past week) after receiving RT than before dental clearance. While emotional functioning improved, cognitive functioning declined after dental clearance and RT. This may be due to increased psychological stress, treatment burden, or physical and nutritional changes during this phase. These outcomes are in accordance with other studies, where emotional functioning is at its lowest before RT treatment [34, 35]. A potential reason for the improvement of emotional functioning might be due to psychological adaptation and coping. For example, participating in the treatment decision-making process has been demonstrated to positively influence patients’ ability to cope with the disease [36]. A possible explanation for diminished emotional well-being prior to treatment may stem from the fact that, shortly after being diagnosed with HNC, emotional problems may become more noticeable in the lives of patients [35]. Another remarkable finding in our study is that HNC patients experience more financial difficulties after the placement of CDs than after receiving RT, on average 24.8 and 2.8 months post-RT, respectively. These findings are in accordance with the studies by Raggini et al. and Ma et al. [37, 38]. The financial distress could be caused by out-of-pocket expenses, such as travel and transportation, parking and taxi fares, dental treatment after RT, and medical bills [37].

One limitation of this study is that not all questionnaires were available at each timepoint. This missing data could be due to non-compliance in completing questionnaires throughout the cancer trajectory, lack of documentation on the type of oral rehabilitation, or patient death. If the characteristics of participants with missing data differed from those with complete data, this could introduce bias, and our final results may not well represent the target population. Additionally, the questionnaires may not adequately capture HRQoL related to oral functioning during oral rehabilitation, which limited our ability to assess these specific aspects and led to a focus on the side effects of RT treatment. More information may have been provided by the additional use of a questionnaire specifically assessing the impact of oral rehabilitation in HNC patients, such as the Liverpool Oral Rehabilitation Questionnaire version 3.0 (LORQv3) or the Vanderbilt Head and Neck Symptom Survey (VHNSS) version 2.0 [39, 40]. The use of these questionnaires could have provided more information about oral functioning in this specific patient group. Another limitation is that the patients who were included in the final analysis may not be well representative to the target population based on Table 1. For example, 60.7% of the patients included in the analysis had a tumor in oropharynx, while only 36.7% of the patients who were excluded from the analysis had the tumor in the same site. Therefore, our final results require further validation in more representative populations. While this treatment pathway reflects standard practice in our center, it is broadly consistent with protocols followed in other European institutions. However, inter-institutional and international variations may influence the generalizability of our findings. Another limitation concerns the documentation of adverse events. As this was a retrospective study, with part of the data derived from handwritten medical records, adverse events were not consistently recorded. Moreover, adverse outcomes were not a primary focus of this study, which may have further limited their identification. Notably, in the four patients who received dental implants, no adverse events were reported. After oral rehabilitation with IODs, patients still reported higher mean scores for problems with taste and smell, dry mouth, sticky saliva, and dependence of tube feeding compared to before dental clearance. Similar to our findings, a study by So et al. reported persisting symptoms including dry mouth, sticky saliva, and changes in taste and smell up to 12 months after completion of RT [41]. Other authors also reported a long-term dependence on nutritional supplements and tube feeding and problems with the sense of smell and taste [26–28]. Furthermore, studies suggest that IODs have a more positive impact on oral functioning and HRQoL than CDs [42, 43]. However, in our study, scores at *T*_3_ were only presented as descriptive statistics due to a small sample size and insufficient data.

Patients experienced an extensive oral rehabilitation time: on average, the TORT between dental clearance and oral rehabilitation with CDs was approximately 13.5 months. For the group of five patients who received IODs, the average TORT was 29.7 months. These extended waiting periods between procedures may have been due to the time required for collaboration and communication between the different medical and dental specialists or a temporary lost to follow-up. Although the timing of oral rehabilitation is generally based on surgical and clinical guidelines, patient-specific variables such as recovery rate, tolerance to RT, and the severity of side effects can significantly affect the actual timing of prosthetic treatment. As optimization of the care trajectory might help to increase QoL in this vulnerable patient population, further research is required to determine the impact of a shorter TORT on the QoL in HNC patients. One of the treatment options to reduce TORT in this vulnerable patient group could include virtual surgical planning, combining a guided bone reduction of the mandibular alveolar process, immediate dental implant placement, and restoration using a prefabricated bar and placement of the overdenture [44]. Oral rehabilitation involving IODs could be beneficial for this specific patient group, particularly considering that irradiated edentulous HNC patients experience enhanced prosthesis retention with dental implants [45]. While immediate reconstruction techniques such as jaw-in-a-day are gaining traction in surgically treated HNC patients, these protocols are not applicable to patients undergoing radiotherapy without surgery. For this group, timely and structured prosthetic rehabilitation remains essential to improving QoL.

Our study population consisted of RT-treated HNC patients who had received full dental clearance. Because of the small sample size and amount of missing data, HRQoL measurements may not be completely representative for the entire population of HNC patients and therefore the results should be interpreted carefully. Nevertheless, given the limited available literature on oral rehabilitation in HNC patients undergoing full dental clearance, we believe this study could significantly contribute to the existing body of knowledge, highlighting optimal oral rehabilitation strategies, improving the HRQoL for HNC patients, and ultimately guiding clinicians in providing more effective and personalized care in such critical cases. However, prospective research with a larger sample size and supervised questionnaire completion is required to ensure more consistency and reliability of the findings.

## Conclusion

Three months after receiving RT, patients reported significantly better emotional functioning compared to their status before dental clearance. However, they also experienced a higher prevalence of issues related to taste and smell, dry mouth, sticky saliva, reliance on nutritional supplements, tube feeding, and weight gain. Additionally, following RT, patients faced limitations in their role and cognitive functions, such as difficulties in performing work or household tasks, and challenges with concentration or memory. Furthermore, patients reported an increase in financial problems after receiving CDs. Patients experienced long waiting periods between dental clearance and oral rehabilitation with CDs. Prospective research on a larger cohort is required to obtain more reliable numbers regarding the HRQoL and to determine whether shorter TORT, including IODs, would have a positive impact on HNC patients’ HRQoL. In addition, oral health-related questionnaires specifically assessing the impact of oral rehabilitation on the HRQoL, such as the VHNSS 2.0, should be implemented in measuring patient-reported outcome measures.

## Data Availability

The data supporting this study are summarized in tables, and figures represent individual data. They are available upon request from the corresponding author.
